# Accurate Detection of Dobutamine-induced Haemodynamic Changes by Kino-Cardiography: A Randomised Double-Blind Placebo-Controlled Validation Study

**DOI:** 10.1038/s41598-019-46823-3

**Published:** 2019-07-19

**Authors:** Amin Hossein, Daniela Corina Mirica, Jérémy Rabineau, José Ignacio Del Rio, Sofia Morra, Damien Gorlier, Antoine Nonclercq, Philippe van de Borne, Pierre-François Migeotte

**Affiliations:** 1 LPHYS, Université Libre de Bruxelles, Bruxelles, Belgium; 2 Department of Cardiology, Erasme Hospital, Université Libre de Bruxelles, Bruxelles, Belgium; 3 BEAMS, Université Libre de Bruxelles, Bruxelles, Belgium

**Keywords:** Cardiovascular biology, Biomedical engineering, Cardiovascular biology

## Abstract

Non-invasive remote detection of cardiac and blood displacements is an important topic in cardiac telemedicine. Here we propose kino-cardiography (KCG), a non-invasive technique based on measurement of body vibrations produced by myocardial contraction and blood flow through the cardiac chambers and major vessels. KCG is based on ballistocardiography and measures 12 degrees-of-freedom (DOF) of body motion. We tested the hypothesis that KCG reliably assesses dobutamine-induced haemodynamic changes in healthy subjects. Using a randomized double-blinded placebo-controlled crossover study design, dobutamine and placebo were infused to 34 volunteers (25 ± 2 years, BMI 22 ± 2 kg/m², 18 females). Baseline recordings were followed by 3 sessions of increasing doses of dobutamine (5, 10, 20 μg/kg.min) or saline solution. During each session, stroke volume (SV) and cardiac output (CO) were determined by echocardiography and followed by a 90 s KCG recording. Measured linear accelerations and angular velocities were used to compute total Kinetic energy (iK) and power (Pmax). KCG sorted dobutamine infusion vs. placebo with 96.9% accuracy. Increases in SV and CO were correlated to iK (r = +0.71 and r = +0.8, respectively, p < 0.0001). Kino-cardiography, with 12-DOF, allows detecting dobutamine-induced haemodynamic changes with a high accuracy and present a major improvement over single axis ballistocardiography or seismocardiography.

## Introduction

The main non-invasive modalities for assessment of cardiac function include ECG, echocardiography (Echo) and cardiac magnetic resonance (cMR). For cardiac contractility assessment, cMR is considered to be the gold standard. However, Echo remains the most commonly used technique in daily clinical practice. Whereas portable and wearable devices can be used to monitor ECG continuously, no such devices exist for home monitoring of cardiac mechanics.

Ballistocardiography (BCG) and seismocardiography (SCG) are lesser-known techniques for assessment of the inotropic state based on measurement of body movements induced by cardiac contraction and blood flow in the cardiac chambers and major vessels. The relationship between cardiac contractility in the broad sense and the ballistic signals was identified, and extensively studied, by Starr in the twentieth century^1^.

The smart-phone industry has driven the innovation of tiny accelerometers using micro-electro-mechanical-systems (MEMS). The availability of this sensitive and affordable technology has generated tremendous interest and opportunity among the biomedical engineering community in development of novel, non-invasive, portable and wearable cardiac monitoring devices^2^.

While BCG primarily measures overall body recoil in response to blood ejection from the heart and pulsatile blood flow in the aorta and its main branches, SCG records precordial accelerations or vibrations of the chest that result from the complex series of movements caused by myocardial contraction. Both BCG and SCG can be measured by MEMS accelerometers or piezoelectric force sensing devices^2^.

There are multiple applications for wearable continuous heart monitors. However, to our knowledge, there is no marketable medical device for non-invasive, automated, inotropic state monitoring. In 2015, we introduced *multidimensional kineticardiography* (MKCG)^3^, which we here renamed Kino-cardiography (KCG). This is a subject-specific calibrated combination of linear and rotational SCG and BCG techniques. KCG measures a total of 12 degrees-of-freedom (DOF) of body motion providing a robust beat-by-beat index of total kinetic energy transferred to the body by the force of cardiac contraction. Since then, the technique of measuring rotational components (gyrocardiography) and the linear and rotational components altogether measured by smartphone (mechanocardiography) have been investigated by others^4,5^. Here, we validate our prototype system– the Kino-cardiograph, which was awarded the EHRA Inventors Award in 2017. KCG has the advantage of combining and integrating various features of gyrocardiography^4^ with the ballistocardiogram-weighing scale system of Inan *et al*.^6^

Dobutamine is a β-adrenergic receptor agonist commonly used for cardiac haemodynamic support and in echocardiography stress testing. We introduced calibrated KCG kinetic energy (iK) and power (Pmax) transferred to the body by cardiac contraction, which are scalar metrics based on 6-DOF measures. Hereafter, the iK and Pmax will be both used as metrics to identify the changes of the physiological status of the subjects.

Our hypothesis was that KCG, with iK and Pmax, is more reliable than conventional single axis SCG and BCG to detect dobutamine-induced haemodynamic changes in healthy volunteers. To the best of our knowledge, this is the first introduction of KCG with its validation through a randomized, double-blind, placebo-controlled, crossover study. iK and Pmax were compared against echocardiography.

## Methods

### Participants and Protocol

The protocol consisted of a randomized, double-blind, placebo-controlled, crossover study in healthy adult subjects between 18 and 50 years of age. Volunteers had no history of cardiac disease, did not smoke, and had BMIs between 20 and 25 kg/m^2^. None took drugs or medications. The study protocol complied with the Declaration of Helsinki, was approved by the local Ethics Committee (Hôpital Erasme – CCB: B406201630013) and is registered on ClinicalTrials.org (April 11, 2017), identifier *NCT03107351*. The prototype device used in this clinical trial was authorized by the Belgian Federal Agency for Medicine and Health Products (FAMHP). Written informed consent was obtained from each subject.

After a baseline recording was obtained, subjects received increasing doses of dobutamine, a β-adrenergic agonist, to increase contractility, heart rate, and cardiac output^7^.

We assumed that cardiac contractility measured by KCG would increase by over 10% for the smallest dose of dobutamine, similar to its effect on stroke volume^8^. Power analysis demonstrated that to achieve 80% power at α = 0.05, at least 32 subjects were needed. Therefore, 36 subjects were recruited to account for possible dropouts and/or technical failures. For the final analysis, 34 participants completed all Echo, ECG, and KCG acquisitions (there were 2 dropouts).

Randomization was performed by random draw and determined that 18 participants would receive dobutamine infusion first (group 1), and 16 participants would receive saline infusion first (group 2). Dobutamine and placebo were administered intravenously through a peripheral arm vein. All measures were performed on subjects lying on the same hospital bed in the left lateral recumbent position.

**Figure 1:**
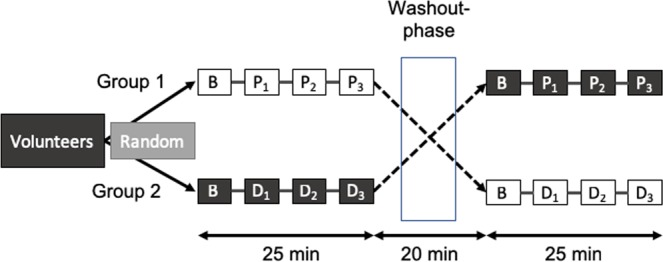
Randomised crossover design of the study. The study population was randomly assigned to one of two experimental groups. B represents baseline period, *P*_1_, *P*_2_ and *P*_3_ are periods of placebo infusion, and *D*_1_, *D*_2_ and *D*_3_ are periods of increasing doses of dobutamine infusion. The washout phase lasted at least 20 minutes.

Each subject underwent two sessions (Fig. 1), with each session subdivided into four identical sequences: baseline measurements prior to infusion followed by three infusion periods of 5, 10, 20 µg/kg.min of dobutamine or saline solution. Each sequence began with a 2 min infusion, then Echo was performed for 2 min and was immediately followed by 90 s of KCG recording. In total, there were eight Echo and KCG measurements: two in the basal state and three each under infusion of dobutamine or normal saline. A washout phase of at least 20 min followed the termination of the first session (Fig. 1). To minimise bias, subjects and operators were both blinded to the sequence of drug administration. A random code was assigned to all KCG and Echo recording sequences and data were analysed in a fully blinded manner as to the sequence and session of the coded recording.

### Kino-cardiography acquisition

**Figure 2:**
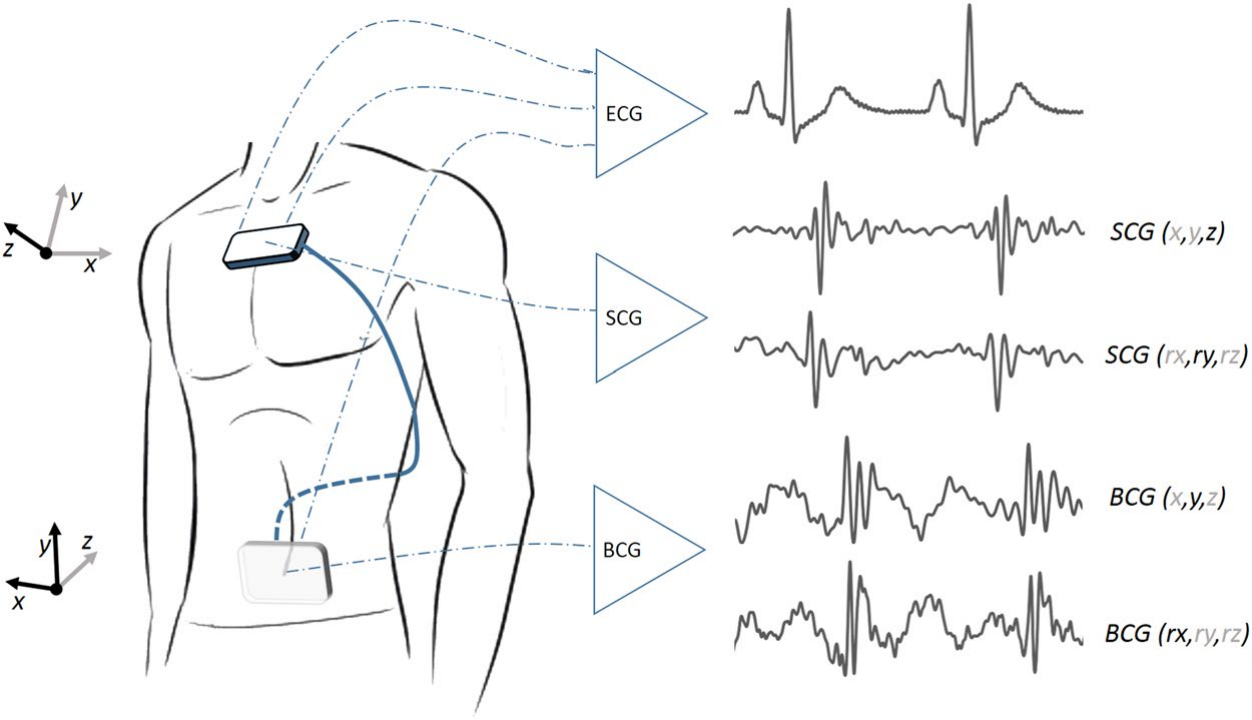
Kino-cardiograph device measuring 2-lead ECG, 6-DOF seismocardiography (SCG) and 6-DOF ballistocardiography (BCG). The standard axis system is used for BCG and SCG: BCG x, y, and z are respectively left-to-right, caudocranial, and ventrodorsal axes; SGC x, y, and z are respectively right-to-left, caudocranial, and dorsoventral axes.

The Kino-cardiograph is a wearable device with two detectors, one of which is placed over the lumbar region close to the subject’s centre of mass and the other close to the heart over the subclavicular sternum (Fig. 2). Each detector contains a MEMS accelerometer and gyroscope sensor (LSM6DSL, STMicroelectronics) and is attached to the body with standard sticky gel electrodes: the lumbar detector is further secured in place with an elastic band. The Kino-cardiograph is controlled with a smartphone or a tablet connected via Bluetooth and collects a two-lead ECG at 200 Hz (ADS1292R, AD Instruments) together with 3-DOF linear (LIN) accelerations and 3-DOF rotational (ROT) angular velocities from the sternum (SCG) and the lumbar region (BCG) (Fig. 2). While BCG measures overall body accelerations, the SCG sensor mainly records local chest wall vibration. In brief, a total of 12-DOF linear acceleration and angular velocity signals are recorded at 50 Hz. A 25 Hz hardware low-pass filter is applied. Standard nomenclature^2^ was used: for BCG signals, *x* is the lateral (left-to-right) axis, *y* is the longitudinal body (caudocranial) axis, and *z* is the anteroposterior (ventrodorsal) axis; for SCG signals, the *z*-axis points in the opposite direction (dorsoventral) and the *x*-axis right-to-left (Fig. 2). More details regarding this method are available in previous publications^3,9,10^.

### Kino-cardiography data analysis

SCG and BCG acceleration and velocity data were calibrated according to constructor datasheets. To ensure synchronicity in processing, data were up-sampled to 1 kHz and low-pass filtered (100 Hz for ECG). The linear accelerations and angular velocities of SCG and BCG data were then processed further. Linear accelerations $(\overrightarrow{a})$ first undergo single-time integration to provide velocity $(\overrightarrow{v})$. Combining these vector components with parameters of Newtonian mechanics, a series of scalar parameters are computed. From the linear components we get:

1 $$\overrightarrow{F}(t)=m.\overrightarrow{a}(t)$$

2 $${K}_{Lin}(t)=\frac{1}{2}m({v}_{x}^{2}(t)+{v}_{y}^{2}(t)+{v}_{z}^{2}(t))$$

3 $${P}_{Lin}(t)=\overrightarrow{F}(t).\overrightarrow{v}(t)$$

where *m* is the mass of the subject, *K*_*Lin*_ is the linear kinetic energy, *v*_*x*_,*v*_*y*_,*v*_*z*_ are components of the velocity vector $\overrightarrow{v}$, $\overrightarrow{F}$ is the force vector, and ${P}_{Lin}$ is the linear power. In addition to the linear components, there are also rotational components of acceleration. If we denote:

4 $$\overrightarrow{\tau }(t)=I\cdot \overrightarrow{\alpha }(t)$$

5 $${K}_{Rot}(t)=\frac{1}{2}({I}_{xx}{\omega }_{x}^{2}(t)+{I}_{yy}{\omega }_{y}^{2}(t)+{I}_{zz}{\omega }_{z}^{2}(t))$$

6 $${P}_{Rot}(t)=\overrightarrow{\tau }(t).\overrightarrow{\omega }(t)$$

where $\overrightarrow{\tau }$ is the torque of force related to the momentum of inertia and $\overrightarrow{\alpha }$ is the angular acceleration, ${K}_{Rot}$ is the rotational kinetic energy, ${I}_{xx}$, $\,{I}_{yy}$, $\,{I}_{zz}$ are the orthogonal components of the moment of inertia, $\overrightarrow{\omega }$ is angular velocity (whose components are ${\omega }_{x}$, $\,{\omega }_{y}$ and $\,{\omega }_{z}$), and ${P}_{Rot}$ is rotational power. To compute the rotational parameters of torque, rotational kinetic energy, and rotational power defined by Eqs (4–6), we use a model of human body moments of inertia calculated at the centre-of-mass along anatomical axes (${I}_{xx}$, $\,{I}_{yy}$, $\,{I}_{zz}$) that takes into account the height and weight of a subject^11^. The hypothesis of orthogonal symmetry to coronal and sagittal planes is made. This model assumes a simple relationship independent of the age, sex, and ethnicity of the subject.

Fiducial point detection was performed on the ECG signal, where R waves were identified and classified as sinus rhythm or abnormal beats. A RR interval time series analysis and classification procedure was used to exclude premature-ventricular contractions and non-sinus rhythm disturbances. Additionally, we applied an outlier detection on beats that would generate too large energies, possibly due to involuntary movement of the subject such as coughing or deglutition or movements of the extremities. When the integral of ${K}^{SCG}$ or ${K}^{BCG}$ of a heartbeat was higher than 5 times the median of the respective kinetic energy of the 5 previous beats, the heartbeat was classified as abnormal. 2409 beats were affected on a total of 30091 beats. Thus 8% of heartbeats were excluded from the analysis. Ensemble averaging (EA) of signals *K*_*Lin*_, *P*_*Lin*_, *K*_*Rot*_ and *P*_*Rot*_ was performed on all normal beats.

The EA signals of variables *K*_*Lin*_, and *K*_*Rot*_ (Eqs (7 and 8)) were integrated on the interval from R wave to the next R wave minus 150 ms, in order to exclude the energy from the next beat P-R interval. The integration interval described herebefore is denoted CC_EA_ . This was performed for both SCG and BCG signals, generating the respective parameters $i{K}_{Lin}^{SCG}$, $i{K}_{Rot}^{SCG},i{K}_{Lin}^{BCG}\,{\rm{a}}{\rm{n}}{\rm{d}}\,i{K}_{Rot}^{BCG}$ for each record for all respiration phases. Maximum values of the power (*P*_*Lin*_ and *P*_*Rot*_) of the EA signals (Eqs (9 and 10)) were also computed for both sensors, generating $P{max}_{Lin}^{SCG}$, $P{max}_{Rot}^{SCG},P{max}_{Lin}^{BCG}\,{\rm{a}}{\rm{n}}{\rm{d}}\,P{max}_{Rot}^{BCG}$.

7 $$i{K}_{Lin}={\int }_{C{C}_{EA}}{K}_{Lin}(t).dt$$

8 $$i{K}_{Rot}={\int }_{{CC}_{EA}}{K}_{Rot}(t).dt$$

9 $$P{max}_{Lin}=\,max({P}_{Lin}(t))$$

10 $$P{max}_{Rot}=\,max({P}_{Rot}(t))$$

By summing the linear and rotational kinetic energy values, a measure of the total cardiac energy and power transmitted to the body by cardiac contraction was obtained.

To our knowledge, this is the first report of KCG combining linear and rotational components of body acceleration to measure the energy produced by cardiac contraction and blood flow within large arteries as an innovative index of cardiac inotropic response.

### Echo-cardiogram measurements and data analysis

Echo recordings and analyses were performed with the GE Vivid E95 by a cardiologist (DM) specialised in non-invasive imaging. Left Ventricular Outflow Tract Diameter (LVOTD), Velocity Time Integral (VTI), Velocity Time Integral Maximum Value (VTI MAX), stroke volume (SV), and cardiac output (CO) were computed. The LVOTD was measured in the parasternal long axis view during systole. LVOT VTI was traced based on the pulsed wave Doppler in the apical long axis. SV was calculated according to Eq. (11).

11 $$SV=\pi {(\frac{LVOTD}{2})}^{2}\ast VTI$$

CO was obtained by multiplying SV by heart rate (HR). Computations of LVOTD, VTI, VTI MAX, SV, and CO were performed in a blinded manner by three different observers (AH, JR, DG). A coefficient of inter-operator variability (CV) was obtained through two-factor ANOVA with fixed effects as described previously^12^. The CVs of VTI, VTI MAX, SV and CO were 3.0%, 2.6%, 6.7%, and 6.9%, respectively, which is comparable to the inter-observer variability benchmarks of the European Recommendations for Echocardiographic requirements^13^.

### Analysis

#### Statistical analysis

Intra-subject Classification: All data analyses were performed off-line using a proprietary software toolbox developed by our team under Matlab (Mathworks Inc.®).

Based on Eqs (7)–(10), scalar parameters $i{K}^{SCG}$, $i{K}^{BCG},P{max}^{SCG}\,{\rm{a}}{\rm{n}}{\rm{d}}\,P{max}^{BCG}$ were calculated from the session and sequence recordings of each subject. The maximum amplitude of the SCG linear dorso-ventral axis $(SC{G}_{Lin}^{z})$, of the SCG rotational caudocranial axis $(SC{G}_{Rot}^{y})$ and the maximum amplitude of the BCG linear caudocranial axis $(BC{G}_{Lin}^{y})$ were also calculated for comparison, as they are the most studied SCG and BCG axes in literature^2^. The blinded analysis team sorted the 8 recordings of each subject in descending order of magnitude of total heart kinetic energy (iK) or heart power (Pmax), metrics that combine linear and rotational parameters as in Eqs (12–15).

12 $$i{K}_{Tot}^{SCG}=(i{K}_{Lin}^{SCG}+i{K}_{Rot}^{SCG})$$

13 $$i{K}_{Tot}^{BCG}=(i{K}_{Lin}^{BCG}+i{K}_{Rot}^{BCG})$$

14 $$P{max}_{Tot}^{BCG}=(P{max}_{Lin}^{BCG}+P{max}_{Rot}^{BCG})$$

15 $$P{max}_{Tot}^{SCG}=(P{max}_{Lin}^{SCG}+P{max}_{Rot}^{SCG})$$

The three highest ranked iK and Pmax metrics for each subject were then assumed to be representative of the sequence with 20 µg/kg.min, 10 µg/kg.min, and 5 µg/kg.min dobutamine infusion sequence, respectively. The remaining five recordings were not attributed any particular sequence since they were assumed to correspond either to placebo or baseline recordings.

Inter-subject classification: Further analyses were conducted and the automated classifications of dobutamine level of all sequence recordings based on iK and Pmax parameters was assessed. Since dobutamine is known to have a chronotropic effect at high concentration^8^, two additional metrics accounting for heart rate were included: $i{K}_{Tot}^{SCG}\ast HR$ and $i{K}_{Tot}^{BCG}\ast HR$. For each subject, average values of SV, CO, VTI MAX and VTI from Echo and average $P{max}_{Lin}^{SCG}$, $P{max}_{Rot}^{SCG},P{max}_{Lin}^{BCG},P{max}_{Rot}^{BCG}$, $i{K}_{Lin}^{SCG}$, $i{K}_{Rot}^{SCG},i{K}_{Lin}^{BCG},i{K}_{Rot}^{BCG}$, $i{K}_{Tot}^{SCG}$, $i{K}_{Tot}^{BCG}$, $i{K}_{Tot}^{SCG}\ast HR$ and $i{K}_{Tot}^{BCG}\ast HR$ were calculated for each dose level of dobutamine or placebo as well as baselines. As the two groups are not paired, baseline characteristics of group 1 and group 2 were compared by a two-sample t-test in case of normal distribution or a Mann-Whitney U test in case of non-normal distribution. Echo and KCG parameters were entered into a linear mixed effects model together with subject sex and the interaction of the dose level of infusion with drugs administered (dobutamine or placebo) as fixed parameters. Group and subject baselines were included in the model as random effects (random intercept model). The homoscedasticity hypothesis was verified by multiple regression. Statistical significance was set at α = 0.05 (two-tailed hypothesis tests). Echo and KCG parameters were compared among dose levels, including baselines, by a paired t-test in case of normal distribution or Wilcoxon signed rank test in case of non-normal distribution. Lilliesfors test was used to test if the difference between sample populations compared was normally distributed. Bonferroni correction was applied to account for multiple comparisons and to generate 95% confidence intervals. Pearson correlation coefficient (*r*) was computed to assess the linear relationships between SV, CO, and $i{K}_{Tot}^{SCG}$, $i{K}_{Tot}^{BCG},i{K}_{Tot}^{SCG}\ast HR$ and $i{K}_{Tot}^{BCG}\ast HR$.

## Results

Baseline characteristics, Echo parameters, and KCG parameters of the entire study population as well as group 1 and group 2 cohorts separately are presented in Table 1. The linear accelerations and angular velocities of both chest and lower-back sensors were computed to get the metrics iK and Pmax. 47.1% of the participants were males, the mean age was 25 years (±1.72), the mean BMI was 22.48 kg/ m² (±2.07), while their mean SV and CO were respectively 62.78 ml (±13.79) and 4.32 l/min (±0.1). $i{K}_{Tot}^{SCG}$ and $i{K}_{Tot}^{BCG}$ were respectively 0.1 mJ.s (±0.09) and 0.01 mJ.s (±0.006). Group 1 and 2 showed to be different regarding the variables $i{K}_{Tot}^{SCG}$, $i{K}_{Tot}^{BCG}$ and, $P{max}_{Tot}^{SCG}\,$and sex distribution.

**Table 1:** Demographic parameters and baseline measurements of echo-cardiographic parameters and kino-cardiographic parameters.

Parameter	Study cohort	Group 1 (N = 16)	Group 2 (N = 18)
Sex (% male)	47.1%*	25%	66.7%
Age (years)	25 ± 1.7	25 ± 1.8	25 ± 1.7
BMI (kg/m2)	22.5 ± 2.1	22.1 ± 1.9	22.9 ± 2.2
SV (ml)	62.8 ± 13.8	59.3 ± 10.0	67.2 ± 15.8
CO (l/min)	4.3 ± 1.0	3.9 ± 0.9	4.7 ± 1.1
VTI (cm)	19.9 ± 2.6	20.1 ± 2.9	20.1 ± 2.1
VTI MAX (m/s)	0.98 ± 0.14	0.97 ± 0.17	1.02 ± 0.12
HR (bpm)	71.6 ± 12.0	68.9 ± 8.5	72.9 ± 11.6
$i{K}_{Tot}^{SCG}$ (mJ.s)	0.10 ± 0.09*	0.05 ± 0.02	0.16 ± 0.12
$i{K}_{Tot}^{BCG}$ (mJ.s)	0.010 ± 0.006*	0.008 ± 0.005	0.015 ± 0.006
$P{max}_{Tot}^{SCG}$ (mJ/s)	21.1 ± 17.4*	14.9 ± 16.5	28.0 ± 17.3
$P{max}_{Tot}^{BCG}$ (mJ/s)	0.87 ± 0.69	0.938 ± 0.81	0.84 ± 0.62

Group 1 corresponds to subjects receiving dobutamine then crossing over to placebo. Group 2 received placebo first and crossed over to dobutamine. Means and standard deviations are shown for continuous variables and percentages are shown for categorical variables. Parameters showing a difference between baseline characteristics of group 1 and group 2 are denoted with an asterisk (*P < 0.05).

**Figure 3:**
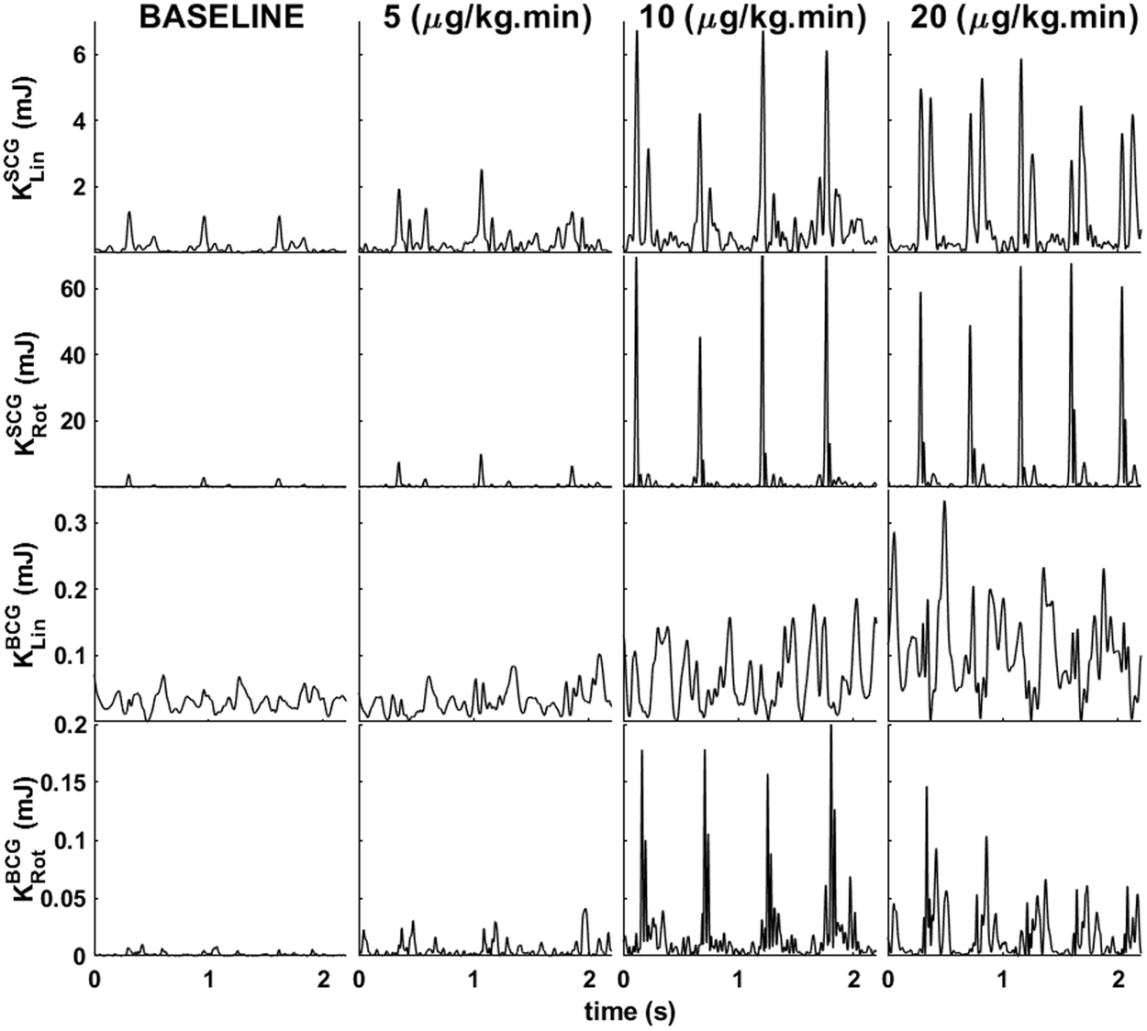
Waveform of (from top to bottom) SCG linear kinetic energy, SCG rotational kinetic energy, BCG linear kinetic energy, and BCG rotational kinetic energy for a representative subject at baseline (left) and then infused with increasing doses of dobutamine (5, 10, 20 μg/kg.min).

Figure 3 illustrates linear and rotational kinetic energy for SCG and BCG for a representative subject during baseline and with increasing doses of dobutamine (5, 10, and 20 μg/kg.min).

### Intra-subject record classification

For each subject $SC{G}_{Lin}^{z}$, $SC{G}_{Rot}^{y}$, $BC{G}_{Lin}^{y}$, $i{K}_{Tot}^{SCG}$ (and $i{K}_{Lin}^{SCG}$, $i{K}_{Rot}^{SCG}$ separately), $i{K}_{Tot}^{BCG}$ (and $i{K}_{Lin}^{BCG}$, $i{K}_{Rot}^{BCG}$separately), $P{max}_{Tot}^{SCG}$, and $P{max}_{Tot}^{BCG}$ were sorted in descending order of magnitude; the three highest values were then assumed to correspond to the dobutamine sequences. A true positive (TP) was defined as correct assignment of a recording made during infusion of dobutamine (5, 10, or 20 μg/kg.min) as among the three highest iK or Pmax values. A true negative (TN) was defined as assignment of a recording made at baseline or during infusion of placebo as among the five lower iK or Pmax values. False negatives (FN) and false positives (FP) were respectively classification of a recording made during dobutamine infusion as among the five lowest values or a recording made during baseline or placebo infusion as among the three highest values. Accuracy, sensitivity, and specificity were calculated as described previously^14^.

Among the 34 subjects, $i{K}_{Rot}^{SCG}$ was associated with a sensitivity of 96% and a specificity of 97.5% when all dobutamine sequences were pooled. $P{max}_{Tot}^{SCG}$ showed a sensitivity of 93.9% and a specificity of 96.2%. SCG kinetic energy and power showed higher sensitivity and specificity than BCG. SV analysed in the same manner had a sensitivity of 93.9% and a specificity of 96.2%. The presented multi-dimensional iK and Pmax based methodology improved the classification accuracy by 3%, 5.5% and 6% when compared to the use of single axes metrics, respectively ${{\rm{SCG}}}_{{\rm{Lin}}}^{{\rm{z}}}$, ${{\rm{SCG}}}_{{\rm{Rot}}}^{{\rm{y}}}$ and ${{\rm{BCG}}}_{{\rm{Lin}}}^{{\rm{y}}}$. Table 2 summarises the accuracy, sensitivity, and specificity for all the parameters considered. The respiration phase is a covariate which has no influence on the classifications.

**Table 2:** Classification of records within each subject based on SV, LVOT VMAX and KCG parameters.

Metric	Accuracy (%)	Sensitivity (%)	Specificity (%)
SV	95.3	93.9	96.2
LVOT VMAX	96.1	95.0	96.8
$SC{G}_{Lin}^{z}$	89.8	86.9	91.7
$i{K}_{Lin}^{SCG}$	89.8	86.9	91.7
$SC{G}_{Rot}^{y}$	89.1	85.9	91.1
$i{K}_{Rot}^{SCG}$	96.9	96	97.5
$i{K}_{Tot}^{SCG}$	93	90.9	94.3
$BC{G}_{Lin}^{y}$	87.5	83.8	89.9
$i{K}_{Lin}^{BCG}$	88.3	84.9	90.5
$i{K}_{Rot}^{BCG}$	88.4	84.9	91
$i{K}_{Tot}^{BCG}$	90.6	87.9	92.4
$P{max}_{Tot}^{BCG}$	90.6	87.9	92.4
$P{max}_{Tot}^{SCG}$	95.3	93.9	96.2

A true positive (TP) corresponds to a recording made during dobutamine infusion (5, 10, or 20 μg/kg.min) classified among the three highest values of the metric. Accuracy, sensitivity and specificity are calculated following the standard.

### Inter-subject record classification

The linear mixed effects model showed significant differences between dobutamine and placebo for all Echo and KCG parameters (*P* < 0.0001) while no significant differences were observed among placebo sequences. In addition, there was no crossover effect for the group on any Echo or KCG parameter.

Table 3 summarises the effects of dobutamine infusion on KCG and echocardiographic parameters. SV, VTI, and VTI MAX mean values increased from baseline to 5 μg/kg.min dobutamine to 10 μg/kg.min dobutamine, with smaller increases seen at 20 μg/kg.min dobutamine. Contrariwise, HR did not change from baseline to 5 μg/kg.min dobutamine but did increase at 10 and 20 μg/kg.min dobutamine. ${{\rm{i}}{\rm{K}}}_{{\rm{T}}{\rm{o}}{\rm{t}}}^{{\rm{B}}{\rm{C}}{\rm{G}}}$ and ${{\rm{i}}{\rm{K}}}_{{\rm{T}}{\rm{o}}{\rm{t}}}^{{\rm{S}}{\rm{C}}{\rm{G}}}$ increased in a similar pattern as SV, without marked increases from 10 to 20 μg/kg.min dobutamine.

**Table 3:** KCG and Echo measurements for each perfusion level (5, 10, and 20 μg/kg.min) of dobutamine.

Parameter (Mean+- STD)	Baseline	5 µg/kg.min	10 µg/kg.min	20 µg/kg.min
SV (ml)	61.2 ± 12.0	71.8 ± 13.0^‡^	82.5 ± 19.0^‡^	84.7 ± 18.0
CO (l/min)	4.1 ± 0.8	4.7 ± 0.8^‡^	6.4 ± 1.0^‡^	8.8 ± 1.9^‡^
VTI (cm)	20.2 ± 2.6	24.3 ± 3.3^‡^	27.7 ± 2.8^‡^	28.1 ± 3.3
VTI MAX (m/s)	0.98 ± 0.13	1.26 ± 0.24^‡^	1.72 ± 0.22^‡^	1.85 ± 0.25^†^
HR (bpm)	70.6 ± 10.3	72.4 ± 10.4	87.3 ± 16.5^‡^	115.2 ± 21.6^‡^
$P{max}_{Tot}^{SCG}$ (mJ/s)	17.8 ± 12.0	96.6 ± 82.0^‡^	200 ± 158^†^	320.1 ± 289
$P{max}_{Tot}^{BCG}$ (mJ/s)	0.8 ± 0.6	3.5 ± 2.7^‡^	4.8 ± 2.8*	6.8 ± 4.7
$i{K}_{Tot}^{SCG}$ (mJ.s)	0.09 ± 0.05	0.23 ± 0.17^‡^	0.36 ± 0.20^†^	0.38 ± 0.20
$i{K}_{Tot}^{BCG}$ (mJ.s)	0.010 ± 0.006	0.018 ± 0.007*	0.025 ± 0.010^†^	0.030 ± 0.010
$i{K}_{Tot}^{SCG}$ * HR (mJ.s/min)	5.7 ± 3.6	16.4 ± 13.2^‡^	31.0 ± 18.6^‡^	42.3 ± 29.6
$i{K}_{Tot}^{BCG}$ * HR (mJ.s/min)	0.68 ± 0.41	1.30 ± 0.61*	2.0 ± 0.92^‡^	3.4 ± 1.9^‡^

KCG and Echo parameters (Mean ± standard deviation) during the baseline and for each concentration level perfusion (5, 10, and 20 μg/kg.min) of Dobutamine. Paired comparison for each KCG and Echo parameters to compare each level of perfusion with the previous level at 5% of significance. *P < 0.005; ^†^P < 0.001; ^‡^P < 0.0001.

Paired t-tests were used to compare parameters between adjacent sequences. Dobutamine (5 μg/kg.min) resulted in increases in all examined KCG and echocardiographic parameters from baseline except for HR. Similar increases, including increases in HR were observed between 5 μg/kg.min and 10 μg/kg.min dobutamine (p < 0.005). Only HR, CO, and VTI MAX from Echo and $i{K}_{Tot}^{BCG}\,\ast \,HR$ from KCG increased from 10 μg/kg.min to 20 μg/kg.min dobutamine. No significant differences in any KCG or echocardiographic parameters were observed between placebo infusions and baseline.

**Figure 4:**
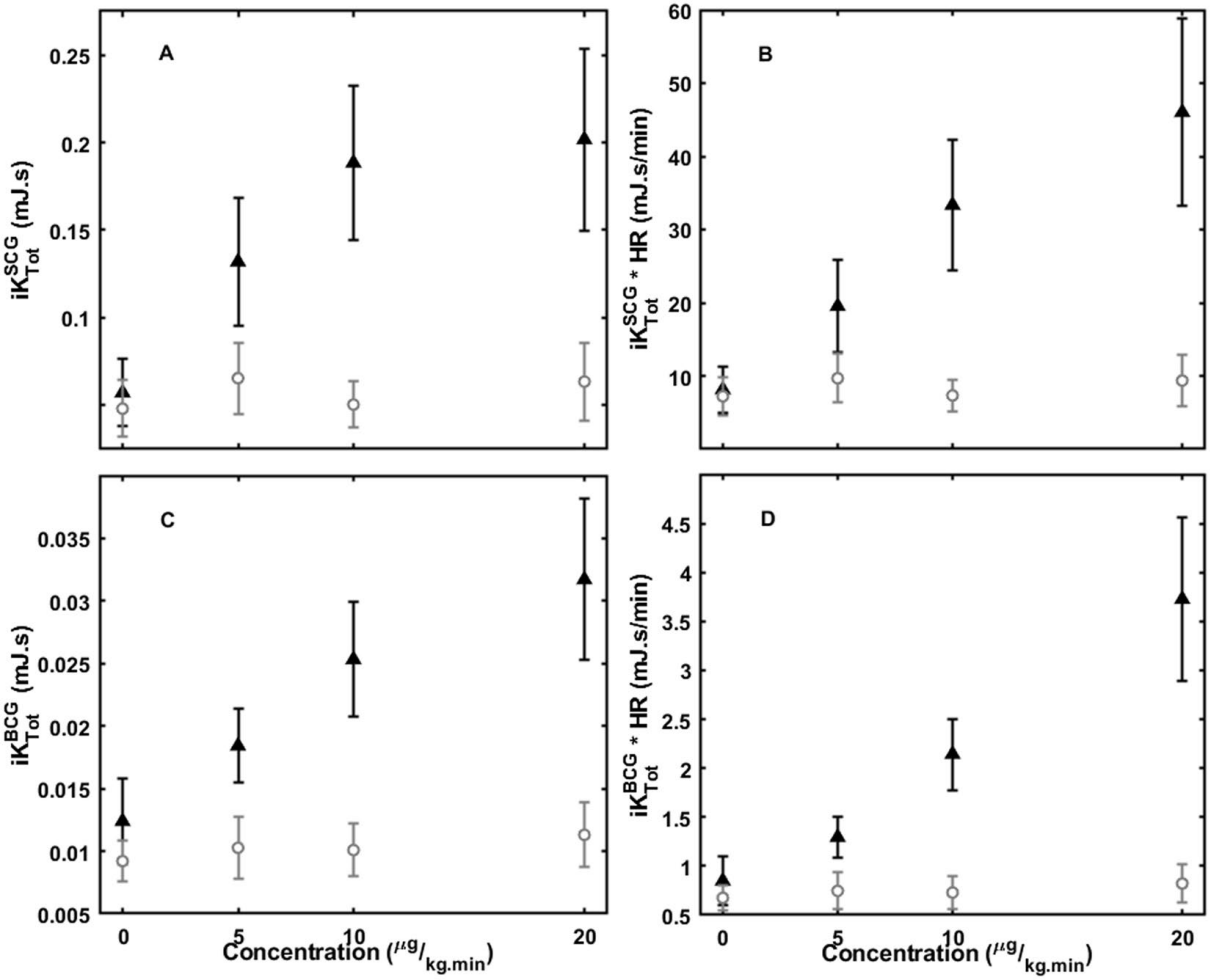
KCG parameters (means and 95% CI): (**A**) $i{K}_{Tot}^{SCG}$, (**B**) $i{K}_{Tot}^{SCG}\,\ast \,HR$, (**C**) $i{K}_{Tot}^{BCG}$, (**D**) $i{K}_{Tot}^{BCG}\,\ast \,HR$ at baseline and at increasing doses of dobutamine or placebo (▴ Dobutamine; ⚬ Placebo).

Figure 4 shows the trends (means and 95% confidence intervals) in KCG parameters $i{K}_{Tot}^{BCG},i{K}_{Tot}^{SCG}$, $i{K}_{Tot}^{BCG}\ast HR$, and $i{K}_{Tot}^{SCG}\,\ast \,HR$ with increasing levels of dobutamine and placebo infusion. $i{K}_{Tot}^{BCG}$ distinguished baseline, 5 μg/kg.min, and 10 μg/kg.min dobutamine dose levels. Multiplying this metric by HR further distinguished 20 μg/kg.min dobutamine from the 10 μg/kg.min dose level.

#### Comparison with echocardiography

**Figure 5:**
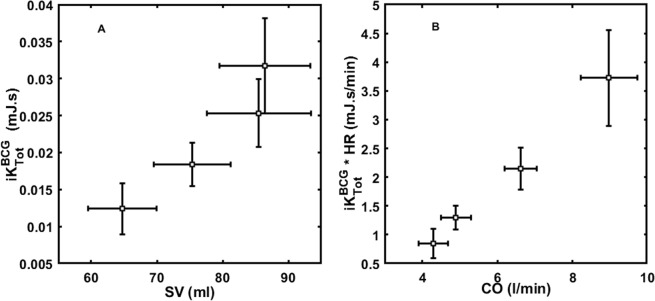
Mean and 95% CI of (**A**) $i{K}_{Tot}^{BCG}$ and SV, (**B**) $i{K}_{Tot}^{BCG}\,\ast \,HR$ and CO at baseline and increasing doses of dobutamine (5, 10, and 20 μg/kg.min). $i{K}_{Tot}^{BCG}$ and SV distinguished baseline, 5 μg/kg.min, and 10 μg/kg.min dobutamine dose levels, but not the 10 μg/kg.min to 20 μg/kg.min dobutamine dose levels. $i{K}_{Tot}^{BCG}\,\ast \,HR$ and CO distinguished baseline, 5 μg/kg.min, 10 μg/kg.min, and 10 μg/kg.min dobutamine dose levels.

CO and $i{K}_{Tot}^{BCG}\,\ast \,HR$ were positively correlated (*r* = 0.8; p < 0.0001), as well as SV and $i{K}_{Tot}^{BCG}$ (*r* = 0.71; p < 0.0001) (Fig. 5). $i{K}_{Tot}^{SCG}\,\ast \,HR$ and $i{K}_{Tot}^{SCG}$ were also positively correlated with CO (*r* = 0.63; p < 0.0001) and SV (*r* = 0.51; p < 0.0001), respectively. $i{K}_{Lin}^{BCG}$ was positively correlated with SV (*r* = 0.73; p < 0.0001). Single axis metrics ($SC{G}_{Lin}^{z}$, $SC{G}_{Rot}^{y}$, $BC{G}_{Lin}^{y}$) were less correlated with SV (r = 0.38, r = 0.40, r = 0.57, respectively; p < 0.0001). The respiration phase is a covariate which does not impact the correlations of iK and Pmax with SV and CO.

## Discussion

### Major findings

This study demonstrates that kino-cardiography, specifically combining calibrated ballisto- and seismocardiographic techniques by means of non-invasive accelerometers and gyroscopes placed over the thoracic and lumbar regions, provides highly selective and specific assessments of dobutamine-induced haemodynamic changes in healthy subjects. We introduced new scalar metrics KCG Kinetic Energy (iK) and KCG Power (Pmax), as innovative parameters for non-invasive estimation of cardiac contractility in routine clinical practice.

${{\rm{P}}{\rm{m}}{\rm{a}}{\rm{x}}}_{{\rm{T}}{\rm{o}}{\rm{t}}}^{{\rm{S}}{\rm{C}}{\rm{G}}}$ and ${{\rm{P}}{\rm{m}}{\rm{a}}{\rm{x}}}_{{\rm{T}}{\rm{o}}{\rm{t}}}^{{\rm{B}}{\rm{C}}{\rm{G}}}$ (mJ/s) are the combination of maximum linear and rotational power detected by SCG and BCG, respectively. They are indicative of the total maximum power generated by the myocardium contraction that is transmitted to the chest wall and to the body at each cardiac cycle. ${{\rm{P}}{\rm{m}}{\rm{a}}{\rm{x}}}_{{\rm{T}}{\rm{o}}{\rm{t}}}^{{\rm{S}}{\rm{C}}{\rm{G}}}\,$and ${{\rm{P}}{\rm{m}}{\rm{a}}{\rm{x}}}_{{\rm{T}}{\rm{o}}{\rm{t}}}^{{\rm{B}}{\rm{C}}{\rm{G}}}$ increase from baseline levels when dobutamine is infused at 5 μg/kg.min and from 5 μg/kg.min to 10 μg/kg.min dobutamine. ${{\rm{i}}{\rm{K}}}_{{\rm{R}}{\rm{o}}{\rm{t}}}^{{\rm{S}}{\rm{C}}{\rm{G}}}$ classified sequence recordings with respect to dobutamine infusions with a high degree of accuracy, sensitivity, and specificity (≥96%). We showed that the calibrated iK and Pmax, which are based on 6-DOF signals, improved the classification sensitivity by more than 5% over the single axis metrics. Furthermore, these 6-DOF metrics present a higher correlation with SV, r = 0.71 (p < 0.0001) than single axis metrics r = 0.57 (p < 0.0001).

${{\rm{i}}{\rm{K}}}_{{\rm{T}}{\rm{o}}{\rm{t}}}^{{\rm{S}}{\rm{C}}{\rm{G}}}$ (mJ.s) and ${{\rm{i}}{\rm{K}}}_{{\rm{T}}{\rm{o}}{\rm{t}}}^{{\rm{B}}{\rm{C}}{\rm{G}}}$ (mJ.s) represent the sum of linear and rotational kinetic energy from SCG and BCG signals, respectively. They are indicative of the total kinetic energy generated by the myocardial contraction that is transmitted to the chest wall and to the body at each cardiac cycle. These parameters also increase from baseline levels when dobutamine is infused at 5 μg/kg.min and from 5 μg/kg.min to 10 μg/kg.min dobutamine. Both ${{\rm{i}}{\rm{K}}}_{{\rm{T}}{\rm{o}}{\rm{t}}}^{{\rm{S}}{\rm{C}}{\rm{G}}}$ and ${{\rm{i}}{\rm{K}}}_{{\rm{T}}{\rm{o}}{\rm{t}}}^{{\rm{B}}{\rm{C}}{\rm{G}}}$ are positively correlated with SV (*r* = 0.51 and *r* = 0.7, respectively). When multiplied by heart rate, these parameters corresponds to estimates of the total kinetic energy generated and transmitted by the heart per minute. We found that $i{K}_{Tot}^{SCG}\,\ast \,HR$ and $i{K}_{Tot}^{BCG}\,\ast \,HR$ were also positively correlated with CO (*r* = 0.63 and *r* = 0.8, respectively). As CO quantifies the volume of blood delivered by the heart per unit time, iK * HR quantifies the amount of kinetic energy produced and transmitted by the myocardial contraction per unit time. $i{K}_{Tot}^{BCG}\,\ast \,HR$ increases with each increasing dobutamine levels, including from 10 μg/kg.min to 20 μg/kg.min dobutamine perfusion level, while remaining at lower stable levels at baseline and during placebo-infusion sequences.

Thus, by computing KCG Kinetic Energy and KCG Power based on calibrated SCG and BCG data, it is possible to detect pharmacologically-induced increases in cardiac contractility. This is a proof of concept of their capacity to follow changes in cardiac contractility. The haemodynamic changes assessed by echocardiography and induced by dobutamine in this study were induced for the only purpose to test the hypothesis that kino-cardiography can reliably detect haemodynamic changes in normal volunteers. Furthermore, we showed that iK and Pmax correlation with conventional echocardiography (SV and CO) were better than the ones with known single axis SCG or BCG metrics.

$i{K}_{Tot}^{BCG}$ and SV plateaued at 10 μg/kg.min dobutamine; this effect was not seen with CO or $i{K}_{Tot}^{BCG}\,\ast \,HR$. This is consistent with results of previous studies, where the effect of increasing dobutamine concentrations (5, 10, 20, 30, and 40 μg/kg.min) in subjects with normal cardiac wall motion was investigated through Echo measurements of SV and CO^8^. HR increase was shown to compensate for SV stagnation above 10 μg/kg.min dobutamine infusion. The increase in HR seen at higher dobutamine doses reduced time for ventricular filling and contributed to the reduction in left ventricular volume and SV. Conversely, the BCG signal is mainly generated by slight variations in the position of the centre of mass, which results from the principle of conservation of momentum. The BCG changes recorded in this study are reactions to pulsatility and blood circulation anisotropy modifications in the arterial tree induced by dobutamine^2^. The increase in SV, in agreement with the Starling mechanisms, contributes to the increase in $i{K}_{Tot}^{BCG}$ via increased mass of blood ejected and to increase blood ejection velocity; this results in a strong correlation between SV and $i{K}_{Tot}^{BCG}.$ SCG signals are produced by both cardiac motion and blood flow in the aorta via energy transmission to the chest wall. $i{K}_{Tot}^{SCG}$ increases with dobutamine concentration are primarily due to stronger cardiac contractions, and perhaps to a lesser degree due to increased SV. The genesis of the BCG and SCG signals is different. BCG records the accelerations generated from the recoil forces in response to blood ejection into the great vessels while the SCG records local vibrations generated from cardiac contraction and transmitted to the chest wall. This supports the idea to acquire the BCG for global haemodynamic and SCG for local cardiac mechanical activity assessment.

Previous studies have highlighted the potential of BCG and SCG in providing reliable information on the contractility status of the heart. Even though using different acquisition systems such as force plate BCG or scale BCG^15,16^, different reference acquired signals, such as BCG specific peaks interval^15,17^ or root-mean-squared BCG power^18^ and different physiological reference metrics, such as PEP^15^ or dP/dt_max_^16^ as surrogate of SV, the results of the present study are in hands with the previous ones^15,18,19^.

Despite the fact that we present here some conclusive correlations between iK/Pmax and CO or SV, the aim of the study was not to claim that these parameters could be an absolute measure of CO or SV. We tried rather to show that they can be used to follow or monitor changes in CO or SV relatively to a predefined state. This could have some applications in the field of cardiac telemonitoring.

These parameters might, indeed, offer additional and complementary information regarding cardiac haemodynamics to traditional echocardiographic signals, which remains the primary diagnostic technique in current clinical practice. For instance, given its ability to correctly classify different stages of increased cardiac contractility, KCG might help monitor heart failure patients during cardiac rehabilitation. Other research teams are also pursuing similar research aims. Inan *et al*.^20^ presented a method based on fusion of three normalised linear SCG axes and use of a graph similarity score (GSS) and found significant differences in GSS between compensated and decompensated heart failure patients. Mechanical parameters extracted from KCG and GSS-derived models are more robust against motion artefacts and other disturbances, as they do not require accurate event-based detection of peaks upon which most other BCG/SCG-based methods rely. Moreover, these metrics are automatically computed and do not necessitate the intervention of an operator. Thus, they may be particularly suitable for real-time analysis and telemonitoring applications. Correct orientation and positioning of the sensors are often discussed^21,22^. With introduction of iK and Pmax metrics, the orientation issue becomes less relevant, as cardiac activity is integrated over all dimensions in scalar parameters that are independent of the frame of reference, and thus independent on sensor orientation. Furthermore, we could expect less inter and intra subject variability as it accounts for all possible transfer of energy between different measurement axes which is a possible explanation for the poor correlation obtained by the single axis metrics. The effect of the position of the sensor (e.g. on the sternum or the cardiac apex) on computed iK and Pmax may require further investigation.

### Strengths and limitations

We used an ensemble averaged beat on each recording to compute KCG parameters and compare them to Echo parameters, however KCG is also suitable to compute beat-by-beat variations of iK and Pmax and for real-time analysis of such parameters. This could allow assessment of changes in heart contractility depending on respiration phases, cardiac arrhythmias, and ectopic beats among other factors.

In order to allow the transmission of data by wireless Bluetooth low energy, the sampling frequency of the SCG and BCG sensors was set to 50 Hz and is close to the theoretical limit as SCG and BCG signals are known to be signals between 1 and 20 Hz^2^. Before conducting this study, we performed additional testing (unpublished observations) on a series of 12 subjects where the same measures were performed with two 3D linear accelerometers from different manufacturers with similar characteristics placed on the same location but sampled at 1000 and 50 Hz respectively. The 50 Hz data were interpolated (cubic spline interpolation) and up-sampled to 1 kHz for comparison. From these two series of acceleration data, the same algorithms were applied to compute iK and Pmax. A Bland-Altman analysis showed that the computed iK are in close agreement (97.19% limits of agreement, +− 2 standard deviation), more details can be found in the supplementary material.

Randomisation of test subjects was performed to prevent accidental selection bias and subjects were randomly attributed to group 1 (receiving dobutamine sequences first) and group 2 (receiving placebo sequences first). Despite this procedure, and probably due to the limited number of subjects included in each group, this random allocation resulted in slightly imbalanced groups, resulting in some baseline differences between groups. However, most importantly and thanks to the crossover study design, this randomisation bias did not affect our results as it was shown by the statistical analysis which failed to identify any significant carry-over effects.

One additional limitation is that Echo and KCG recordings were not acquired simultaneously. Measurements were separated by about 2 minutes. We thus compared data from slightly different inotropic states that may have negatively affected correlation analysis. Our study was designed in this way to minimise the risk that, if acquired simultaneously, iatrogenic movements induced by the echocardiographer could have disrupted the KCG signals. A further consideration is that the inertia model used in this study to compute rotational energy was established using a regression model based on the height and weight of test subjects. This model does not consider morphologic differences between sexes or ethnicity. We performed a sensitivity analysis of the model parameters uncertainty to provide robust estimates of momentum of inertia and their impact on the computed iK and Pmax, this can be found in the supplementary material. Furthermore, we used the same model for both BCG and SCG. Although model parameters are well suited for BCG acquisition, they are obviously a rough approximation for the SCG components which deserve thus further improvements. Whereas the BCG sensor is positioned near the centre of mass, representing movements of the whole body due to cardiac activity, and thus suitable to calibration by overall body mass and moments of inertia, SCG signals are generated by movements in the precordial region. As suggested by Yang *et al*.^23^, the mass and inertia model used for SCG could be subject-independent and more related to the acquisition device itself. Otherwise, the SCG model might require adaptation to the mass of the body parts that are accelerated. Lastly, the moments of inertia may need adjustment to a new centre of rotation. However, this would only affect the absolute value of the computed kinetic energy and not relative changes, which should remain in the same proportions. Thus, while the absolute inter-subject values would be affected, relative changes observed between subjects would not be.

Furthermore, KCG parameters are relying on signal amplitude to generate iK and Pmax. Thus, changes in the mechanical coupling of the sensors to the body could impact these metrics. However, this effect was not observed in this study, as baseline and placebo measurement remained stable and without any significant differences.

CO and SV were measured by echocardiography, which is known to be operator-dependent and therefore not perfect gold standards. To mitigate this limitation, Echo acquisition was performed by a single highly experienced clinician and all Echo analyses were performed by three different analysts blinded to subject, session, and sequence. Further studies in patients requiring invasive catheter-based measurements for clinical care may provide even stronger correlations between changes in haemodynamic status and iK and Pmax than in the present trial.

It should also be remembered that KCG recordings were obtained in recumbent subjects at rest and devoid of cardiac rhythm disturbances. Movement artefacts in patients with cognitive impairment or neurological disease or in patients with prevalent arrhythmia such as atrial fibrillation might provide additional computational challenges. Lastly, we were able to achieve sizeable increases in cardiac output that were readily detectable by KCG. Studies testing whether this novel methodology is equally sensitive to clinically relevant reductions in cardiac function in patients are still needed.

## Conclusion

This study demonstrates that kino-cardiography and its kinetic energy and power scalar parameters, based on 12-DOF signals, present important improvements over the more common single axis seismo- and ballisto-cardiography. It was shown that kino-cardiography detects dobutamine-induced haemodynamic changes in healthy subjects with a high degree of accuracy, sensitivity and specificity (≥94%). Furthermore, the observed increases in KCG parameters were strongly correlated with stroke volume and cardiac output measured by echocardiography. Recordings of body vibrations produced by myocardial contraction and blood flow using micro-electro-mechanical systems may become a powerful tool to non-invasively monitor cardiac inotropic activity and provide complementary information to standard techniques currently used in clinical practice.

## Supplementary information

Supplementary Material

## Data Availability

The datasets generated and analysed support the findings of this study are available from the authors upon reasonable request.

## References

[1] Starr I (1958). The relation of the ballistocardiogram to cardiac function. Am. J. Cardiol..

[2] Inan OT (2015). Ballistocardiography and seismocardiography: a review of recent advances. IEEE J. Biomed. Health Inform..

[3] Scherz W. Daniel, Soria Morillo Luis Miguel, Seepold Ralf (2016). Biological Data Tracing and Pattern Recognition in Real-time. XIV Mediterranean Conference on Medical and Biological Engineering and Computing 2016.

[4] Jafari Tadi M (2017). Gyrocardiography: A New Non-invasive Monitoring Method for the Assessment of Cardiac Mechanics and the Estimation of Hemodynamic Variables. Sci. Rep..

[5] Iftikhar Z (2018). Multiclass Classifier based Cardiovascular Condition Detection Using Smartphone Mechanocardiography. Sci. Rep..

[6] Inan OT, Etemadi M, Wiard RM, Giovangrandi L, Kovacs GTA (2009). Robust ballistocardiogram acquisition for home monitoring. Physiol. Meas..

[7] Mertes H (1993). Symptoms, adverse effects, and complications associated with dobutamine stress echocardiography. Experience in 1118 patients. Circulation.

[8] Pellikka PA (1995). Normal stroke volume and cardiac output response during dobutamine stress echocardiography in subjects without left ventricular wall motion abnormalities. Am. J. Cardiol..

[9] Lejeune L, Caiani EG, Prisk GK, Migeotte P-F (2014). Evaluation of ensemble averaging methods in 3D ballistocardiography. Conf. Proc. Annu. Int. Conf. IEEE Eng. Med. Biol. Soc. IEEE Eng. Med. Biol. Soc. Annu. Conf..

[10] Migeotte P-F (2011). Three dimensional ballistocardiography: methodology and results from microgravity and dry immersion. Conf. Proc. Annu. Int. Conf. IEEE Eng. Med. Biol. Soc. IEEE Eng. Med. Biol. Soc. Annu. Conf..

[11] Matsuo A, Ozawa H, Goda K, Fukunaga T (1995). Moment of inertia of whole body using an oscillating table in adolescent boys. J. Biomech..

[12] Popović ZB, Thomas JD (2017). Assessing observer variability: a user’s guide. Cardiovasc. Diagn. Ther..

[13] Galderisi M (2011). Recommendations of the European Association of Echocardiography How to use echo-Doppler in clinical trials: different modalities for different purposes. Eur. J. Echocardiogr..

[14] Šimundić A-M (2008). Measures of diagnostic accuracy: basic definitions. Med. Biol. Sci..

[15] Ashouri H, Orlandic L, Inan OT (2016). Unobtrusive Estimation of Cardiac Contractility and Stroke Volume Changes Using Ballistocardiogram Measurements on a High Bandwidth Force Plate. Sensors.

[16] Tavakolian, K. *et al*. Myocardial contractility: A seismocardiography approach. In *2012 Annual International Conference of the IEEE Engineering in Medicine and Biology Society* 3801–3804, 10.1109/EMBC.2012.6346795 (2012). 10.1109/EMBC.2012.6346795 23366756

[17] Zhang X (2018). A rapid approach to assess cardiac contractility by ballistocardiogram and electrocardiogram. Biomed. Tech. (Berl).

[18] Inan OT, Etemadi M, Paloma A, Giovangrandi L, Kovacs GTA (2009). Non-invasive cardiac output trending during exercise recovery on a bathroom-scale-based ballistocardiograph. Physiol. Meas..

[19] Etemadi M (2014). Tracking Clinical Status for Heart Failure Patients using Ballistocardiography and Electrocardiography Signal Features. Conf. Proc. Annu. Int. Conf. IEEE Eng. Med. Biol. Soc. IEEE Eng. Med. Biol. Soc. Annu. Conf..

[20] Inan OT (2018). Novel Wearable Seismocardiography and Machine Learning Algorithms Can Assess Clinical Status of Heart Failure Patients. Circ. Heart Fail..

[21] Ashouri H, Inan OT (2017). Automatic Detection of Seismocardiogram Sensor Misplacement for Robust Pre-Ejection Period Estimation in Unsupervised Settings. IEEE Sens. J..

[22] Wiens AD, Etemadi M, Roy S, Klein L, Inan OT (2015). Toward Continuous, Noninvasive Assessment of Ventricular Function and Hemodynamics: Wearable Ballistocardiography. IEEE J. Biomed. Health Inform..

[23] Yang C, Tang S, Tavassolian N (2017). Utilizing Gyroscopes Towards the Automatic Annotation of Seismocardiograms. IEEE Sens. J..

