# Publisher Correction: Accurate Detection of Dobutamine-induced Haemodynamic Changes by Kino-Cardiography: A Randomised Double-Blind Placebo-Controlled Validation Study

**DOI:** 10.1038/s41598-020-61864-9

**Published:** 2020-03-20

**Authors:** Amin Hossein, Daniela Corina Mirica, Jérémy Rabineau, José Ignacio Del Rio, Sofia Morra, Damien Gorlier, Antoine Nonclercq, Philippe van de Borne, Pierre-François Migeotte

**Affiliations:** 1 LPHYS, Université Libre de Bruxelles, Bruxelles, Belgium; 2 Department of Cardiology, Erasme Hospital, Université Libre de Bruxelles, Bruxelles, Belgium; 3 BEAMS, Université Libre de Bruxelles, Bruxelles, Belgium

Correction to: *Scientific Reports* 10.1038/s41598-019-46823-3, published online 19 July 2019

In the original version of this Article, the author Philippe van de Borne was incorrectly indexed. This error has now been corrected.

