# Supplementary material for: Accurate Detection of Dobutamine-induced Haemodynamic Changes by Kino-Cardiography: A Randomised Double-Blind Placebo-Controlled Validation Study
Source: Sci Rep. 2019 Jul 19;9:10479. doi: 10.1038/s41598-019-46823-3 (PMC6642180; doi:10.1038/s41598-019-46823-3)
Supplement: Supplementary file 1 — Supplementary Material [file 41598_2019_46823_MOESM1_ESM.pdf]

SUPPLEMENTARY MATERIAL

Accurate Detection of Dobutamine-induced Haemodynamic Changes by Kino-Cardiography: A Randomised Double-Blind Placebo-Controlled Validation Study

Amin Hossein[1,3], Daniela Corina Mirica[2], Jérémy Rabineau[1], José Ignacio Del Rio[2], Sofia Morra[2], Damien Gorlier[1], Antoine Nonclercq[3], Philippe van de Borne[2*], Pierre-François Migeotte[1*].

[1]LPHYS, Université Libre de Bruxelles, Belgium

[2]Department of Cardiology, Erasme Hospital, Université Libre de Bruxelles, Belgium

[3]BEAMS, Université Libre de Bruxelles, Belgium

[*]Both authors contributed equally
Contact for correspondence:

Hossein Amin
Hôpital Erasme, Cardiology Department
Route de Lennik 808
1070, Anderlecht
Brussels, Belgium
0032484810868
amin.hossein@ulb.ac.be

1. Kino-cardiography Metric Computation

Ensemble averaging (EA) of signals K_{Lin}, P_{Lin}, K_{Rot}, and P_{Rot} was performed on all normal beats. The EA signals of variables K_{Lin}, and K_{Rot} were integrated on the interval from R wave to the next R wave minus 150 ms, in order to exclude the energy from the next beat P-R interval (Figure A). This was performed for both SCG and BCG signals, generating the respective parameters iK_{Lin}^{SCG}, iK_{Rot}^{SCG}, iK_{Lin}^{BCG}, and iK_{Rot}^{BCG} for each record for all respiration phases. Maximum values of the power (P_{Lin} and P_{Rot}) of the EA signals were also computed for both sensors, generating $Pmax_{Lin}^{SCG}$, $Pmax_{Rot}^{SCG}$, $Pmax_{Lin}^{BCG}$, and $Pmax_{Rot}^{BCG}$.

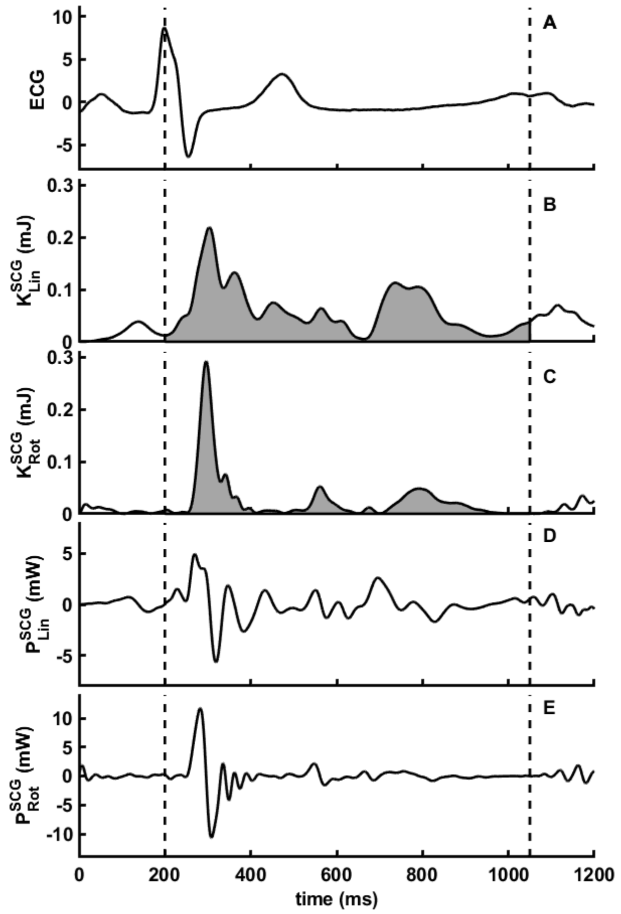

Figure A Ensemble averaged signals of the SCG sensor. The area under highlighted under curves B and C represent respectively iK_{Lin}^{SCG} and iK_{Rot}^{SCG}. The dashed line at the beginning of the signal represents the start of the integration (the ECG-R peak). The dashed line at the end of the signal (next ECG-R peak minus 150 ms) represents the end of the integration.

2. Inertia Model

There is no practical possibility to calculate the principal moments of inertia of the body of each subject. A full-body scan, which is impractical to perform for each

subject, would be such a solution. However, in this work we approximate the subject's moments of inertia through a model based on existing literature [1]. The model is based and the weight and height of each subject to provide an approximate of the moments of inertia.

Moreover, we did test the sensitivity of the current models to slight differences in the input parameters and found e.g. that 10% uncertainty on the parameters of the model would lead respectively to 4%, 6% and 7% of changes in I_{xx}, I_{yy} and I_{zz}, which would lead to less than 7% of error in the K_{rot} parameter. We also used a database containing the weight, the height and the moments of inertia of 66 subjects [2] and compared it to the moments of inertia as they would be calculated by the model that we used in this paper. The results are shown in the figure B (unpublished data). The errors approach 0% for I_{XX} and 10% for I_{YY} and I_{ZZ}. These errors in the estimate would apply all along the recordings on a single subject and would possibly affect the inter-subject variability but not the intra-subject one.

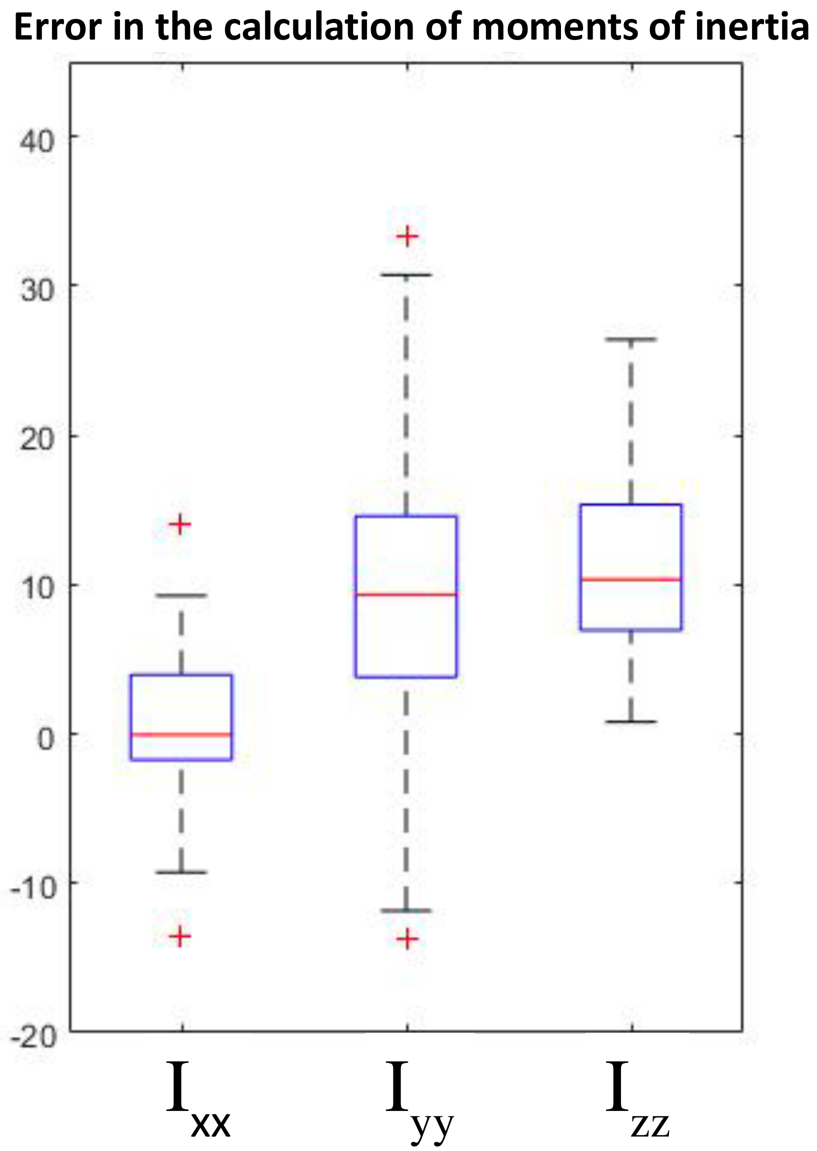

Figure B Error in the moments of inertia computed with the model used in this study with real values. Comparison in 66 subjects.

3. SCG and BCG Sampling Rate Validation

The sampling frequencies of the BCG and SCG signals were set at 50 Hz and are close to the theoretical limit as BCG and SCG are known to be signals between 1 and 20 Hz [3]. Before conducting the study, we performed additional testing (unpublished observations) on a series of 12 subjects where the same measures were performed with 2 accelerometers from different vendors placed on the same location but sampled at 1000 and 50 Hz respectively. The 50 Hz data were interpolated (cubic spline interpolation) and up-sampled to 1 kHz (well above any

theoretical limit) for comparison. From these two series of acceleration data, the same algorithms were applied to compute iK and Pmax. The Bland-Altman plot (see figure C) shows that the computed iK are in close agreement (97.19% limits of agreement, +- 1.96 standard deviation).

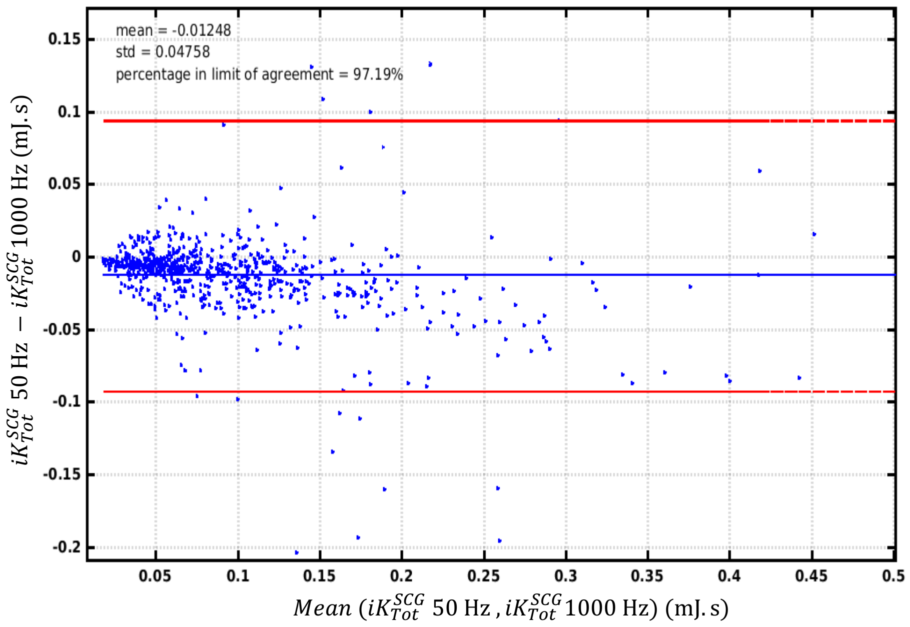

Figure C Bland-Altman plot comparing the computation of iK_{Tot}^{SCG} *on the basis of SCG signals acquired at 50 Hz or at 1000 Hz on 12 subjects.*

References

1. Matsuo, A., Ozawa, H., Goda, K. & Fukunaga, T. Moment of inertia of whole body using an oscillating table in adolescent boys. *J. Biomech.* **28**, 219–223 (1995).
2. Santschi, W. R., DuBois, J. & Omoto, C. *Moments of Inertia and Centers of Gravity of the Living Human Body*. (North American Aviation Inc Los Angeles CA, 1963).
3. Inan, O. T. *et al.* Ballistocardiography and seismocardiography: a review of recent advances. *IEEE J. Biomed. Health Inform.* **19**, 1414–1427 (2015).
